# The launch of the Translational Perioperative and Pain Medicine

**Published:** 2014-07-06

**Authors:** Renyu Liu

**Affiliations:** 1 Department of Anesthesiology and Critical Care, Perelman School of Medicine at the University of Pennsylvania, Philadelphia PA 19104, USA

William T.G. Morton, on October 16, 1846, demonstrated the use of ether anesthesia at the Massachusetts General Hospital. He opened a gateway to the translational medicine in modern anesthesiology and perioperative medicine. However, it took over 100 years before this compound was utilized for anesthetic purposes and eventually, the role of ether in modern medicine was replaced with safer drugs such as sevoflurane, desflurane, etc. Despite these drugs are essential for modern anesthesia, the underlying mechanism of general anesthetics remain elusive(1) and true neurological outcomes are currently a hot topic of debate/research in aged brain and neonatal brain.(2,3) Further studies are warranted and novel anesthetics with minimal side effects are to be discovered and developed.

Morphine is one of the greatest examples in translational pain medicine. Natural morphine can be derived from the opiate, and humans have used opiates dating back to the third-century B.C. However, morphine, the first active alkaloid as a modern medicine, was not discovered and extracted from the opium poppy plant until 1804 in Germany by Friedrich Sertürner, a 20-year-old pharmacy apprentice with limited education. His discovery did not raise immediate scientific and business interests. Therefore, he became both a scientist and entrepreneur to promote his own discovery and business. Twelve years later, in 1817, he and his company marketed morphine for analgesia, and as a “cure” for opium and alcohol addiction. It was quickly revealed that morphine itself is more addictive than opium or alcohol. Sertürner suffered from morphine addiction and chronic depression in later years. He judged himself a failure in his quest to develop a safe and efficient analgesic formula and a means to treat pain without rendering the patient unconscious. The molecular target of morphine was not revealed until 1973, when Candace Pert and Solomon H. Snyder discovered mu opioid receptors using radioactive naloxone binding studies at Johns Hopkins University School of Medicine.(4) The structures of opioid receptors were revealed using crystallography in 2012. (5-7) While it seems that the research related to opioids and opioid receptors had final conclusions, it is actually just the beginning of a golden age from an improved understanding of the structure function relationships. Although it is still unclear why opioids are addictive, scientists continue to search for safe pain medications, and the problems related to opioid abuse were never abated and are actually increasing. To this day, the best medication available for pain management is probably still morphine. Opioids are the preferred drug in pain management during perioperative periods and remain to be the last resort for various chronic and severe pain.

These histories give us great lessons for drug discovery in anesthesiology, perioperative arena, and pain medicine. It is important to note that despite their usage throughout the centuries, mechanisms for both general anesthetics and opioids are not well understood yet. In the case of general anesthetics, no definitive target(s) has been identified yet. And while opioids have many side effects, including fatal respiratory depression and addiction, they are the major medications for severe pain and used frequently during the perioperative period. Thus, while the mechanism is critically important, the lack of understanding of mechanism should not be a reason to delay any development of the new technology for clinical usage. It is also important to note that there is a huge time discrepancy between the initial discovery and the final clinical usage. Certainly, the development of an innovation in medicine is much faster now; however, drug development and various technological developments in medicine are never so complicated due to strict regulations and other barriers, unveiling many layers before the implementation of the new technology. As such, most new technologies must go over multiple “death valleys” before they gain true life in clinical practice.

Since almost all scientific journals focus on academic achievement, papers introducing new technology are not easily accepted and may take numerous revisions and require collection of new data, especially these related to mechanisms. Patent filing may be significantly delayed due to this lengthy publication process. Even after the paper is accepted and the patent is filed, the inventors are generally uncertain how to progress, and the investors, entrepreneurs, and industries do not easily access the critical information until a related paper is published or the patent is open to the public. A platform is needed to reduce the gaps from novel technology to clinical practice. To build a platform, we are launching this journal, Translational Perioperative and Pain Medicine, in an effort to reduce the gaps for any innovative work in perioperative and pain medicine to daily clinical practice. Our major focus is not to seek high impact factor at the cost of delaying the development of novel technology or novel ideas in improving patient care, but to seek faster and higher business success in developing new technology and transpiring knowledge to improve translational research, technology transfer, clinical practice, and patient care in perioperative and pain medicine.

The first issue of the journal will serve as a sample issue to denote the papers that will be accepted, including editorials, research papers, case reports, and scientific or business reviews related to translational medicine.

One important feature of this journal is the significant wisdom and contribution from industries. The board is comprised of physicians, physician scientists, patent lawyers, technology transfer experts, and marketing experts. We hope that this will be an outstanding platform to merge science, business and clinical practice for perioperative and pain medicine. We also strongly believe that it is critical to train the next generation of physicians with business sense, thus, we opened a special resident corner to encourage residents in anesthesiology and various surgical specialties to participate in such efforts. We welcome residents to submit mini-reviews and studies related to new technologies in the perioperative and pain medicine.
